# Epidemiology, Genetics and Epigenetics of Congenital Heart Diseases in Twins

**DOI:** 10.7759/cureus.17253

**Published:** 2021-08-17

**Authors:** Ramya Balasubramanian, Sravya Vuppalapati, Chaithanya Avanthika, Sharan Jhaveri, Nikhil Chowdary Peddi, Sana Ahmed, Apeksha Reddy, Jaskaranpreet Kaur

**Affiliations:** 1 Pediatrics, Smt. Kashibai Navale Medical College, Pune, IND; 2 Pediatrics, People's Education Society Institute of Medical Sciences and Research, Kuppam, IND; 3 Pediatrics, Karnataka Institute of Medical Sciences, Hubli, IND; 4 Internal Medicine, Smt. Nathiba Hargovandas Lakhmichand Municipal Medical College, Ahmedabad, IND; 5 Internal Medicine, Smt. Kashibai Navale Medical College, Pune, IND; 6 Otolaryngology, Dayanand Medical College Ludhiana, Punjab, IND

**Keywords:** pediatric genetics, screening, twin twin transfusion, twins, congenital heart disease, epigenetics, placenta and heart, dichorionic, monochorionic, pediatric cardiology

## Abstract

Congenital heart defects (CHDs) refer to abnormalities in the heart function that arise at the fetal stages. It is the most common birth defect that affects 0.8% of all liveborn infants. There is an increase in the incidence of congenital heart disease in monochorionic twin gestation. A six-fold increase in CHDs exists among monochorionic twins especially in association with twin-twin transfusion syndrome (TTTS) compared to dichorionic twin pregnancy. In this review article, we discussed the epidemiology, the role of genetics like protein-coding genes, epigenetics, placenta, hemodynamics and environmental factors in the etiology of CHD in twins.

We conducted a literature search in PubMed indexed journals using the medical terms "twin pregnancy" and "congenital heart defect" to provide an overview of the uptrend in CHD in twin pregnancies, primarily due to assisted reproductive technologies (ARTs) and multiple other factors. Both the heart and placenta are vascular and share a common development window; therefore, CHD can develop secondary to placental pathologies. Among environmental factors, the strongest association of maternal smoking with CHD has been seen. We studied the causative factors to suggest improvement in echocardiographic skills in case of abnormal findings in twin gestations to decrease the CHD-associated morbidity and mortality, as early diagnosis allows doctors to precisely determine the risk of CHD. Systemic ultrasound scanning with five transverse views is very effective in diagnosing fetal CHD in twin pregnancy. In the case of genetics, prenatal counseling allows the expectant to understand the full ramifications of possible events after the pregnancy. The pathological basis of malformations specific to conjoined twinning and twin reversed arterial perfusion sequence is addressed. Also, there is evidence that folate supplementation may be protective against CHD but more research is needed to clarify the mechanisms.

We concluded from the literature that monochorionic twins are at high risk of CHD. Chorionicity seems to play a more vital role than zygosity. Even the type of heart defect in monochorial twin pregnancies was unique from single, dizygotic, or dichorionic twin pregnancies. We also emphasize improving echocardiographic skills of technicians in referring ART dichorionic twin fetuses with suspicious findings to fetal cardiologists and performing postnatal scans in the case of TTTS. To understand the role of the placenta, making use of newer technologies and examining the placenta both during pregnancy and beyond delivery will play a vital role in understanding the etiology. Even identifying early signals impacting the heart and placental vasculature and correcting them using advanced technology could downtrend the incidence in coming years. Increased maternal age as well as multiple pregnancies increasing the risk of CHD has also been implicated. For more clarity on the role of genetics, the cost of DNA sequencing needs to decrease. This will enable whole-genome sequencing in the future thus helping to discover the gene responsible for CHD ultimately proving beneficial for future generations. For environmental factors, we have to rely on observational studies to assess the risk to the unborn child. There is difficulty in studying natural factors due to the unreliability of exposure to contaminants like pesticides and air pollution.

## Introduction and background

The percentage of twin births in the general population has grown during the previous two centuries. Several embryos are implanted at once to ensure conception in assisted reproductive technologies (ARTs). Thereby, ARTs are most likely to blame for the rise in twin births [1,2]. Overall, advanced maternal age accounts for one-fourth to one-third of the rise in twin pregnancies, while subfertility therapy accounts for the remainder. Although dizygotic (DC) twins appear to constitute the majority of ART-related twinning, higher frequencies of monozygotic (MC) twins (including monochorionic) have also been observed [3]. In general, twins have a greater risk of congenital abnormalities than singletons, especially when it comes to cardiovascular anomalies [2,4].

Ventricular septal defects (VSDs), atrial septal defects (ASDs), tetralogy of Fallot (TOF), and transposition of the major arteries are all cardiac development abnormalities that start early in the fetal development [5]. Congenital heart defects (CHDs) range in severity from mild to severe and complex. ASDs and VSDs are typically asymptomatic until school age or early adulthood. Others such as hypoplastic left heart syndrome, tetralogy of Fallot, and transposition of great arteries can cause cyanosis and inadequate pulmonary and systemic perfusion if adequate surgical treatment is not received [2].

Twin-twin transfusion syndrome (TTTS), which is a common complication in monochorionic twins (sharing a placenta), is primarily linked to the development of CHDs. Unbalanced blood flow from the donor twin to the receiver twin via placental vascular anastomoses causes TTTS, which affects 10%-15% of MC twin pregnancies [6]. Due to this, the risk of CHD in monochorionic (MC) twins is higher than that of dichorionic (DC) twins (who do not share a placenta) [7]. However, in recent years, it has been clear that a diverse set of protein-coding genes, as well as other substances like non-coding RNAs, are important in maintaining a limited and particular pattern of gene expression in the heart, which can be associated with an increased risk of CHDs in twins [8]. This literature review aims to correlate the links between CHDs in twin pregnancies, with the associated genetic and epigenetic risk factors and mechanisms involved.

## Review

Epidemiology

CHDs are the most common human birth defect with a prevalence of 7-9 per 1,000 singleton live births [9,10]. CHDs are much more common in twin pregnancies, with a reported prevalence of 20 in 1000 live births [10]. Monochorionic (MC) twins are at a higher risk compared to dichorionic (DC) twins [10]. In addition, monochorionic/diamniotic (MC/DA) twin pregnancies complicated by TTTS confer a 13- to 14-fold increase in CHD risk compared with the general population [11]. The pathophysiologic mechanisms behind this significant increase in CHD risk have not yet been explored thoroughly. It appears that the interplay of abnormal placentation in conjunction with a genetic predisposition may play some part in this increased risk for CHD [12].

According to international data, about eight million children have been born globally after the birth of the first child Louise Brown in 1978 via ART [13]. The association between MC/DA twin gestation and CHDs has important clinical implications because the number of twin gestations is steadily rising in the United States partly because of the increased use of assisted reproductive technologies. This has increased the rate of both dichorionic and monochorionic twins [14].

The etiology of CHD is hypothesized to be of both genetic and hemodynamic origin [15]. Although twin-twin transfusion in monochorionic twins was identified as an important risk factor for CHD, it does not explain why there would be an increased risk in dichorionic twins. Others hypothesized that placental vascular anastomoses between the monozygotic twins' circulations might lead to fluctuations in blood flow during fetal heart development, causing CHD [16,17]. Literature stated that the etiology of CHD in twins was predominantly hemodynamic as opposed to genetic, which explained why chromosomal anomalies were less common in twins with CHD than singletons [18]. Given that all monochorionic twins are monozygotic and around 10% of dichorionic twins are monozygotic, it explained that although there was an increased risk in both monochorionic and dichorionic twins equally, the magnitude of CHD was greater in monochorionic twins [19]. The increased risk in dichorionic twins was related to the use of ART. In their literature review, Bahtiyar et al. [11] found VSDs to be the most frequent congenital anomaly in the sample of monochorionic/diamniotic twin gestations overall. The most frequent congenital heart defect subtype was coarctation of the aorta [11]. PDA was commonly seen due to prematurity as expected.

The developing embryo can be affected by genetic factors that are inherited or by the environment. Identical twins arise from the same ovum and have identical genetic structures, so their difference was hypothesized to arise from some difference in the environment. Formerly, most congenital defects were thought to have a genetic basis, but environmental factors have recently become increasingly prominent.

The factors affecting normal growth and development leading to CHD can occur as early as the first weeks of life, supported by high rates of cardiac looping and laterality defects in discordant CHD and the higher prevalence of CHD in twins conceived by ART [3,20]. Studies suggested that higher disease prevalence in twins conceived by ART was influenced by gamete manipulation, culture, and embryo implantation in nonfamilial CHD. Although Monochorionic twins share a placenta In later months of gestation, they do not necessarily experience identical growth environments. Anastomoses between fetal circulations in TTTS [21] which has a high frequency of CHD (26%), indicate that hemodynamics plays a critical role in the pathophysiology of discordant CHD. The growth conditions for the recipient and donor twin are significantly different, which subsequently influences cardiac development. A study that analyzed ventricular strain changes in MC twins affected by TTTS shows that the ventricular strain of the recipient at all Quintero stages was reduced compared with donors [22]. In Quintero stages 3 and 4, recipient right ventricular strain was reduced compared with donors [22]. This is due to reduced aortic distensibility in the recipient fetus leading to reduced ventricular strain and impaired filling [22]. The relative risk of right ventricular outflow tract obstruction in MC twins with TTTS is 70 times that of singletons, thus emphasizing the likely importance of hemodynamics on the development of cardiac structures in the second trimester when the heart has fully formed [23].

Even though there is a genetic basis for CHD in twins, the incidence of CHD can be reduced if we know the environmental factors affecting disease causation. For a decade, there has been a decrease in CHD prevalence from a steady upward trend in Europe since the past decade [22] and Canada [24]. The downward trend may be due to increased use of folic acid supplementation in pregnant women, better follow-up of patients with chronic diseases (e.g., diabetes), and reductions in CHD risk factors such as maternal smoking. Therefore, there is an imminent need to study the epidemiology of CHD in twins even further. Studies would help establish the true CHD burden and could improve awareness among the public. They would also be able to provide data to evaluate the impact of interventions in reducing CHD incidence in the developed and developing world that has undergone rapid demographic, economic, and environmental changes.

Monozygotic twins vs. dizygotic twins

Depending on the number of eggs (zygotes) fertilized during pregnancy, two main types of twinning can occur- monozygotic (MZ) and dizygotic (DZ). MZ twins, often known as identical twins are the outcome of a single fertilized egg dividing into distinct embryos during conception. Therefore they are 100% genetically identical. Dizygotic twins, often known as fraternal twins occur when two eggs are released at almost the same time and become fertilized. These eggs then develop into two distinct fetuses because DZ twins are a product of two distinct eggs and are genetically identical, sharing 50% of their genes on average [25].

A higher proportion of monozygotic (MZ) twins are reported to have a single placenta. Stern cites a Steiner table showing that 70% of MZ twins have a single placenta, while only 44% of DZ twins do; perhaps more importantly, that 57% of MZ twins have a single chorion but none of the DZ twins do [19].

Monochorionic placentation is linked to a higher incidence of CHD [3]. In 2007, a systematic review and meta-analysis of four studies found that MC twins have a nine-fold higher risk of CHD than singletons [23]. Altered placentation coupled with inter-twin vascular anastomoses could be a sign of angiogenic factor dysregulation [3]. TTTS has been linked to the development of acquired CHDs in MC twins. The uneven blood flow from one twin to the other through placental vascular anastomoses causes TTTS, affecting 10%-15% of monochorionic twin pregnancies [23].

Furthermore, alterations in fetal hemodynamics could lead to CHD development in these MC twins [3]. However, studies show that monozygotic and dizygotic twins have an increased risk of congenital heart defects than singletons [19]. We conclude that congenital cardiac defects are more likely in twins than in singletons because dizygotic twins are all dichorionic and increased occurrence is not limited to monochorionic twins [19]. As a result, irrespective of chorionicity or zygosity, intrauterine surveillance and a postnatal thorough cardiac evaluation for both twins may be recommended [19].

Gedda claims that cardiac abnormalities are induced by a lack of oxygen caused by the abnormal functioning of the maternal-fetal circulation caused by the type of placentation and that this explains why monozygotic twins have a greater rate of cardiac malformations. According to Polani, increased blood flow could also cause some cardiovascular abnormalities [26]. This proposal acknowledges that hemodynamic forces play a significant role in the normal development of the heart and major arteries and that when these forces diverge from normal, cardiovascular abnormalities can occur. Such differences in twins may be caused by vascular communication between the two twins, which is considerably more common in MZ twins for the reasons stated [26].

Placenta-heart relations

The placenta performs an essential function in fetal health. Since it is not a completely shaped organ at the onset of pregnancy, the placenta needs to meet the enlargement of the fetus to satisfy its crucial function throughout pregnancy. Without a doubt, the placenta and the fetal heart expand in parallel, a phenomenon known as the placenta-heart axis. This leaves the growing heart specifically liable to early placental insufficiency. Both organs share numerous developmental pathways; they are also vulnerable to genetic defects [27,28]. Multiple traces of evidence like genetic [29], hemodynamic, vascular [30] and metabolic, e.g., folate deficiency [27,29] endorse a full-size ability of the placenta to cause non-syndromic CHD [28]. 

The placentas of newborns with CHD are smaller than anticipated and show several vascular abnormalities, with TGA being the most prominent. In mothers sporting a fetus with congenital coronary heart disease(CHD), an imbalance in pro-angiogenic and anti-angiogenic elements in each maternal blood and fetal cord blood is said [31].

Maternal preeclampsia, a medical result of placental vascular dysgenesis, is related to fetal CHD, suggesting a shared pathway of development among those situations, with an aberration in early angiogenesis likely resulting in abnormality of both the placenta and consequently the fetal system [32,33]. Not only is it viable to suspect that the placenta is de-novo abnormally formed in the fetus with CHD but variations in blood flow patterns and perfusion traits unique to the form of heart malformation present may also affect the enlargement and development of the placenta throughout gestation. As an example, particularly low flow or altered oxygen delivery as visible in situations like hypoplastic left heart syndrome [5] or transposition of the great arteries [8], respectively, may also affect placental growth and development, in addition impacting the capability of this vital interface for fetal maturation. 

The placental structure also has an effect on the fetus with CHD and consequently merits exploration. The placenta in CHD has a marked increase in fibrin deposition, reduced terminal villi, and accelerated expression of leptin, an angiogenic and mitogenic hormone produced through the placenta, which may also suggest a shot to make amends for vascular abnormalities [34]. It also suggests the idea of an early, common beginning to abnormal placentation and developmental abnormalities of the fetal system [35]. For example, Chorangiosis-a growth in capillary density in line with placental tissue may also ultimately be a compensatory reaction that can lower average placental vascular resistance by growing the vascular cross-sectional area [2]. Studies investigating the link between placental abnormalities and post-natal effects may also provide perception into the fetal origins of CHD. Doppler-derived volumetric calculations of umbilical venous flow as an absolute measure of placental blood flow, or possibly a relative fraction of average fetal flow are additionally more informative [36,37]. Other methods like improved MRI techniques will help find relations between blood flow, placental structure, and overall performance throughout pregnancy, thus adding critical insights to our current understanding [36,38,39]. Currently, there are no biomarkers available in medical practice for the pre-or post-natal detection of CHDs. 

The placenta is now being recognized to play a “gate-keeper” function in cardiac improvement through a couple of mechanisms [40]. Many gene mutations cause placental morphological abnormalities and congenital heart defects [40]. Folate is understood to be protecting the placental- coronary heart axis in opposition to environmental insults [29]. Several environmental insults are successively acknowledged to play a crucial function in the occasion of non-syndromic CHD. Other greater samples of the function of the placenta in CHD improvement are usually encountered in medical obstetrics. There are two major arteries connecting the placenta to the fetal system, specifically the umbilical arteries. Failure to develop one of the umbilical arteries (single umbilical artery) is well-known to cause an increased risk of numerous CHDs in the fetus [30]. Therefore in conditions with similarly decreased blood supply, e.g. TTTS, a greater than 9-fold growth in CHD is seen. These placental vascular diseases are managed with prenatal laser therapy to reverse the structural abnormalities. This confirms the critical role of placental vascular development in CHD pathogenesis. Further altered methylation of angiogenesis genes can cause CHD like vascular endothelial growth factor (VEGF), which has been related to normal placental angiogenesis [41]. Thus, coordinated development of the placenta and fetal heart and also the consequences for placental involvement in the etiology and pathogenesis of CHD are evident [28].

Genetics

Both conserved genetic alterations and de-novo mutations play pivotal roles in congenital heart diseases. Initially, a genetic component of CHD was implicated in studies concerning the deletion of the 22 q11.2 gene [42]. Many studies have since been undertaken to prove that a strong genetic component in various inherited CHDs exists [43-45]. One can see trends in these genetic disorders, as most diseases have a common pathophysiological origin.

The largest proportion of CHDs is attributed to chromosomal aneuploidies. Hartman showed that Trisomy 21,18 & 13 contributed significantly to the total karyotype yield of CHDs [46]. Moreover, the incidence of CHD in liveborn with these disorders was recorded to be much higher, with 35 to 50% in Down's, and as high as 80% in Trisomy 18 and 13. The evidence of CHDs in Turner syndrome live borns is found to be 33 to 42% as well [46]. Therefore clinicians recommend karyotype analysis in the presence of clinical suspicion of CHD, as aneuploidies have far-reaching complications beyond isolated CHD [47]. 

Like most congenital diseases, deviation from normal embryological development of the heart leads to potential CHD morbidities. Notably, two important players deserve our attention. The first is the NOTCH pathway family of genes responsible for synthesizing proteins that participate in a host of processes- from ventricle development to left-right organization [48] via the activation of the intracellular NOTCH domain. Mutations in this family of genes are responsible for a whole host of CHDs. For instance, mutations in JAG-1 are found in over 90% of patients with Alagille Syndrome [49]. JAG-1 mutations also contribute to isolated CHDs, most commonly found with TOF [49]. 

The second is the Cilia Gene family which is responsible for generating hair-like organelles involved in many physiological processes. It is not surprising that mutations in these genes have far-reaching consequences involving other organ systems, notably the brain [50]. One notable example is Kallman syndrome, which has been linked to 35 genes as of now.

As mentioned above, although conserved genetic mutations are an important cause of CHDs, with individuals with affected siblings and parents found to be at greater risk (more than two times) [51], they make up only 2.2% of all reported CHDs [52]. Moreover, there is strong evidence suggesting that CHD patients have poor reproductive fitness. This leads us to speculate that a majority of CHDs would come from de-novo mutations, a hypothesis tested and proved by a multitude of studies using Whole Exome Sequencing [53]. Many of these de-novo mutations were found in chromatin remodeling genes, which were found in the enhancer and promoter regions of critical developmental genes [54]. 

Epigenetics

An insight into congenital cardiac abnormalities can be gained by analyzing the epigenetic pathways in identical discordant twins [55,56]. Understanding the factors that cause molecular plasticity is difficult but it will add to our present understanding of CHD [56]. In many investigations of the heart's differential methylation profile, the tissue level is used rather than the cell level [57]. However, this is not ideal as analysis at tissue level may overlook numerous differentially methylated genes [57]. This problem is fixed by the single-cell DNA methylation sequencing technique, which helps identify stage-specific issues in signals and regulatory pathways of cardiogenesis [56, 57]. Because of the lack of access to human cardiac tissue during the early stages of cardiogenesis, bulk sequencing investigations (e.g., transcriptomics) have been performed on cardiac tissues obtained from terminated fetuses or surgical samples [56].

In twins with tetralogy of Fallot (TOF), it has been observed that a good number of genes in cardiac tissue have been linked to differential DNA methylation like the NKX2-5 (NK2 Homeobox 5) and HAND1 (Heart And Neural Crest Derivatives Expressed 1) genes [58]. It was noted that there are multiple single differentially methylated CpGs (5'-Cytosine-phosphate-Guanine-3') with more than a 25% difference between the twins [58]. Using DNA methylation profiling technology, Yu et al. (2018) showed that a double outlet right ventricle (DORV) discordant monozygotic twin pair had differentially methylated regions in his study [59].

Multiple DMCs (differentially methylated regions) were discovered in the promoter CGI (CpG islands) of a gene that encodes for the Transcription factor NFATC1 (nuclear factor of activated T cells 1). It is required for normal valve formation in patients with CHD and in the promoter CGI (CpG islands) of TBX20 (T-Box transcription factor 20), which is another cardiac transcription factor seen in different forms of cardiac defects. Various congenital heart defects have been linked to disruptions in these associated cardiac regulation networks [60].

Another study shows that age and Apo-E methylation levels are inversely related and the degree of Apo-E methylation is protective against CHD [61]. A GMDR (generalized multifactor dimensionality reduction) interaction analysis in CHD revealed a link between Apo-E methylation and rs7412 polymorphism, a crucial factor in the formation of types of proteins in Apo-E. This link confirms why Apo-E4 carriers have a higher risk of CHD than Apo-E2 carriers [61]. Some of the hypomethylated maternal genes could result in CHD inheritance in their kids [58]. Current research suggests that histone modification, chromatin remodeling and small non-coding RNA may also have a role in the epigenetics of CHD that are particularly vulnerable to environmental exposures, for e.g., the consequences of disulfiram observed in monochorionic twins with cleft palates and limb abnormalities [58,20]. 

When monozygotic twins with CHD were compared to their non-CHD co-twins, there was evidence of a collection of deregulated miRNAs. These miRNAs' predicted target genes play a role in cardiomyocyte signaling [2]. Based on this evidence, we have come to learn that MiRNAs and DNA methylation are viable indicators for the diagnosis of CHD patients before and after birth [55]. Differential DNA methylation discovered in the placenta and blood of neonate and mother is employed as a diagnostic biomarker [57]. It is now evident that demethylation therapy during pregnancy may help to minimize the risk of CHD. Present DNA demethylation medications work by reducing the total methylation level rather than on methylation of specific genes or regions [57].

## Conclusions

It is a well-known fact that twin gestations have a higher risk of complications than singleton gestations. Evidence suggests that the development of the placenta and heart occurs in a particular sequence with many signals that, when coordinated poorly, give rise to congenital heart diseases. We do not understand how these signals between genetics and placental development cause CHD, which has been the area of focus in molecular genetic sciences research. The majority of monochorionic twin gestations have a higher risk of CHD because of (TTTS), but not limited, as it is also seen in dichorionic twin gestations- particularly because of two placental developments which should be taking place in a synchronized manner with the fetal heart development. Even with immense advances in neonatal science, the etiology of most CHD cases without genetic implication remains a mystery. We recommend future research projects to delve deeper into the environmental factors impacting this disease, as this area can be studied better. Our review mainly focused on epigenetics, genetics and the placental-heart relationship in the disease process of CHDs in twins over the years.

## References

[1] Heino A, Gissler M, Hindori-Mohangoo AD (2016). Variations in multiple birth rates and impact on perinatal outcomes in Europe. PLoS One.

[2] Abu-Halima M, Weidinger J, Poryo M, Henn D, Keller A, Meese E, Abdul-Khaliq H (2019). Micro-RNA signatures in monozygotic twins discordant for congenital heart defects. PLoS One.

[3] Panagiotopoulou O, Fouzas S, Sinopidis X, Mantagos SP, Dimitriou G, Karatza AA (2016). Congenital heart disease in twins: the contribution of type of conception and chorionicity. Int J Cardiol.

[4] Glinianaia SV, Rankin J, Wright C (2008). Congenital anomalies in twins: a register-based study. Hum Reprod.

[5] van der Linde D, Konings EE, Slager MA, Witsenburg M, Helbing WA, Takkenberg JJ, Roos-Hesselink JW (2011). Birth prevalence of congenital heart disease worldwide: a systematic review and meta-analysis. J Am Coll Cardiol.

[6] Lopriore E, Vandenbussche FP, Tiersma ES, de Beaufort AJ, de Leeuw JP (1995). Twin-to-twin transfusion syndrome: new perspectives. J Pediatr.

[7] Best KE, Rankin J (2015). Increased risk of congenital heart disease in twins in the North of England between 1998 and 2010. Heart.

[8] Espinoza-Lewis RA, Wang DZ (2012). MicroRNAs in heart development. Curr Top Dev Biol.

[9] Pinborg A (2005). IVF/ICSI twin pregnancies: risks and prevention. Hum Reprod Update.

[10] Blondel B, Kaminski M (2002). Trends in the occurrence, determinants, and consequences of multiple births. Semin Perinatol.

[11] Bahtiyar MO, Dulay AT, Weeks BP, Friedman AH, Copel JA (2007). Prevalence of congenital heart defects in monochorionic/diamniotic twin gestations: a systematic literature review. J Ultrasound Med.

[12] Bjarnegård M, Enge M, Norlin J (2004). Endothelium-specific ablation of PDGFB leads to pericyte loss and glomerular, cardiac and placental abnormalities. Development.

[13] Zaliska O, Stasiv K, Maksymovych N (2020). The trends of assisted reproductive technologies and cost for ovarian stimulation protocols in Ukraine. Pharmacia.

[14] Toledo MG (2005). Is there increased monozygotic twinning after assisted reproductive technology?. Aust N Z J Obstet Gynaecol.

[15] Bruneau BG (2008). The developmental genetics of congenital heart disease. Nature.

[16] Hall JG (2003). Twinning. Lancet.

[17] Pharoah POD (2005). Causal hypothesis for some congenital anomalies. Twin Res Hum Genetics.

[18] Jones KL, Jones MC, del Campo M (2013). Smith’s recognizable patterns of human malformation. https://www.us.elsevierhealth.com/smiths-recognizable-patterns-of-human-malformation-9781455738113.html.

[19] Herskind AM, Almind Pedersen D, Christensen K (2013). Increased prevalence of congenital heart defects in monozygotic and dizygotic twins. Circulation.

[20] AlRais F, Feldstein VA, Srivastava D, Gosnell K, Moon-Grady AJ (2011). Monochorionic twins discordant for congenital heart disease: a referral center's experience and possible pathophysiologic mechanisms. Prenat Diagn.

[21] Benirschke K (1995). The biology of the twinning process: how placentation influences outcome. Semin Perinatol.

[22] Taylor-Clarke MC, Matsui H, Roughton M, Wimalasundera RC, Gardiner HM (2013). Ventricular strain changes in monochorionic twins with and without twin-to-twin transfusion syndrome. Am J Obstet Gynecol.

[23] Gijtenbeek M, Shirzada MR, Ten Harkel AD, Oepkes D, C Haak M (2019). Congenital heart defects in monochorionic twins: a systematic review and meta-analysis. J Clin Med.

[24] Liu S, Joseph KS, Luo W (2016). Effect of folic acid food fortification in Canada on congenital heart disease subtypes. Circulation.

[25] DiLalla LF, Mullineaux PY, Elam KK (2008). Twins. Encyclopedia of Infant and Early Childhood Development.

[26] Campbell M (1961). Twins and congenital heart disease. Acta Genet Med Gemellol.

[27] Maslen CL (2018). Recent advances in placenta-heart interactions. Front Physiol.

[28] Radhakrishna U, Albayrak S, Zafra R (2019). Placental epigenetics for evaluation of fetal congenital heart defects: Ventricular Septal Defect (VSD). PLoS One.

[29] Linask KK (2013). The heart-placenta axis in the first month of pregnancy: induction and prevention of cardiovascular birth defects. J Pregnancy.

[30] Araujo Júnior E, Palma-Dias R, Martins WP, Reidy K, da Silva Costa F (2015). Congenital heart disease and adverse perinatal outcome in fetuses with confirmed isolated single functioning umbilical artery. J Obstet Gynaecol.

[31] Hajdu J, Beke A, Marton T, Hruby E, Pete B, Papp Z (2006). Congenital heart diseases in twin pregnancies. Fetal Diagn Ther.

[32] Sifrim A, Hitz MP, Wilsdon A (2016). Distinct genetic architectures for syndromic and nonsyndromic congenital heart defects identified by exome sequencing. Nat Genet.

[33] Krützfeldt J, Rajewsky N, Braich R, Rajeev KG, Tuschl T, Manoharan M, Stoffel M (2005). Silencing of microRNAs in vivo with 'antagomirs'. Nature.

[34] van Boven N, Kardys I, van Vark LC (2018). Serially measured circulating microRNAs and adverse clinical outcomes in patients with acute heart failure. Eur J Heart Fail.

[35] Wang K, Zhou LY, Wang JX (2015). E2F1-dependent miR-421 regulates mitochondrial fragmentation and myocardial infarction by targeting Pink1. Nat Commun.

[36] Rychik J, Goff D, McKay E, Mott A, Tian Z, Licht DJ, Gaynor JW (2018). Characterization of the placenta in the newborn with congenital heart disease: distinctions based on type of cardiac malformation. Pediatr Cardiol.

[37] Figueras F, Fernández S, Hernández-Andrade E, Gratacós E (2008). Umbilical venous blood flow measurement: accuracy and reproducibility. Ultrasound Obstet Gynecol.

[38] Andescavage N, Yarish A, Donofrio M (2015). 3-D volumetric MRI evaluation of the placenta in fetuses with complex congenital heart disease. Placenta.

[39] Sun L, Macgowan CK, Sled JG (2015). Reduced fetal cerebral oxygen consumption is associated with smaller brain size in fetuses with congenital heart disease. Circulation.

[40] Camm EJ, Botting KJ, Sferruzzi-Perri AN (2018). Near to one's heart: the intimate relationship between the placenta and fetal heart. Front Physiol.

[41] Sundrani DP, Reddy US, Chavan-Gautam PM, Mehendale SS, Chandak GR, Joshi SR (2014). Altered methylation and expression patterns of genes regulating placental angiogenesis in preterm pregnancy. Reprod Sci.

[42] Goodship J, Cross I, LiLing J, Wren C (1998). A population study of chromosome 22q11 deletions in infancy. Arch Dis Child.

[43] Garg V, Muth AN, Ransom JF (2005). Mutations in NOTCH1 cause aortic valve disease. Nature.

[44] Schott JJ, Benson DW, Basson CT (1998). Congenital heart disease caused by mutations in the transcription factor NKX2-5. Science.

[45] Garg V, Kathiriya IS, Barnes R (2003). GATA4 mutations cause human congenital heart defects and reveal an interaction with TBX5. Nature.

[46] Hartman RJ, Rasmussen SA, Botto LD (2011). The contribution of chromosomal abnormalities to congenital heart defects: a population-based study. Pediatr Cardiol.

[47] Geddes GC, Earing MG (2018). Genetic evaluation of patients with congenital heart disease. Curr Opin Pediatr.

[48] Han P, Bloomekatz J, Ren J (2016). Coordinating cardiomyocyte interactions to direct ventricular chamber morphogenesis. Nature.

[49] Oda T, Elkahloun AG, Pike BL (1997). Mutations in the human Jagged1 gene are responsible for Alagille syndrome. Nat Genet.

[50] Zaidi S, Brueckner M (2017). Genetics and genomics of congenital heart disease. Circ Res.

[51] Murabito JM, Pencina MJ, Nam BH (2005). Sibling cardiovascular disease as a risk factor for cardiovascular disease in middle-aged adults. JAMA.

[52] Øyen N, Poulsen G, Boyd HA, Wohlfahrt J, Jensen PK, Melbye M (2009). Recurrence of congenital heart defects in families. Circulation.

[53] Choi M, Scholl UI, Ji W (2009). Genetic diagnosis by whole exome capture and massively parallel DNA sequencing. Proc Natl Acad Sci U S A.

[54] Zaidi S, Choi M, Wakimoto H (2013). De novo mutations in histone-modifying genes in congenital heart disease. Nature.

[55] Jarrell DK, Lennon ML, Jacot JG (2019). Epigenetics and mechanobiology in heart development and congenital heart disease. Diseases.

[56] Lim TB, Foo SY, Chen CK (2021). The role of epigenetics in congenital heart disease. Genes.

[57] Cao J, Wu Q, Huang Y, Wang L, Su Z, Ye H (2021). The role of DNA methylation in syndromic and non-syndromic congenital heart disease. Clin Epigenetics.

[58] Ghanchi A, Derridj N, Bonnet D, Bertille N, Salomon LJ, Khoshnood B (2020). Children born with congenital heart defects and growth restriction at birth: a systematic review and meta-analysis. Int J Environ Res Public Health.

[59] Matias A, Silva S, Blickstein I (2020). Mechanisms of discordance in monozygotic twins: why and when?. Developmental and Fetal Origins of Differences in Monozygotic Twins.

[60] Grunert M, Appelt S, Grossfeld P, Sperling SR (2020). The needle in the haystack-searching for genetic and epigenetic differences in monozygotic twins discordant for tetralogy of fallot. J Cardiovasc Dev Dis.

[61] Ji H, Zhou C, Pan R (2019). APOE hypermethylation is significantly associated with coronary heart disease in males. Gene.

